# Commentary: Efficacy of Dog Training With and Without Remote Electronic Collars vs. a Focus on Positive Reinforcement

**DOI:** 10.3389/fvets.2021.629746

**Published:** 2021-04-29

**Authors:** Rebecca J. Sargisson, Ian G. McLean

**Affiliations:** ^1^School of Psychology, University of Waikato, Tauranga, New Zealand; ^2^Researcher, Tauranga, New Zealand

**Keywords:** dogs, e-collars, predation, training, punishment

## Introduction

In an experimental analysis of the effectiveness of e-collars, China et al. (1) concluded that “there is no evidence to indicate that E-collar training is necessary” (p. 1). The paper contributes to a wider body of research on the use of e-collars for dog training, much of which is referenced in the paper. In this commentary, we focus on whether the methods and analysis support the findings, point to methodological inconsistencies between this and a companion paper (2), describe concerns with the statistical analysis, and suggest that the conclusions go well beyond the results.

E-collars are commonly used to reduce or prevent canine predation or aggression. With reference to welfare concerns, the justification is that predation behavior is life-threatening for both dog and attacked animal. An example is e-collar training to prevent hunting dogs from attacking kiwi [in New Zealand; (3)]. An intense electric shock is paired with a target stimulus (a stuffed kiwi) to produce a classically conditioned aversive response. The shock is delivered only once or twice, establishing a response that produces reliable avoidance of the target stimulus for up to 3 years (4).

China et al.'s (1) stated aim is to assess “the efficacy of the use of electronic collars to improve recall … and general obedience in dogs compared to training without E-collars” (p. 2). China et al. (1) do not claim to assess the efficacy of e-collars for the prevention of canine attacks. However, the paper may be used by governments to support a ban of e-collars for *all* training purposes, including the prevention of aggression (5).

## Method and Results

There are issues with the experimental design. The “positive reinforcement” group of dogs was trained at a different time, at a different location, and by different trainers, relative to the other two groups. Additionally, assignment to groups was only semi-random; two owners did not want their dogs to be shocked, so two dogs assigned to the shock group were exchanged with two originally assigned to a no-shock group. These issues lower confidence in comparability of the data across groups. The results also depend critically on training outcomes on Day 5 when owners, rather than trainers, trained the dogs. Comparability with the results on earlier days are compromised by this methodological shift.

China et al. (1) report on a re-analysis by new observers of videos previously reported in less detail (2). Data (videos) were recorded over 5 days of training, with analysis and reporting of responses on Days 1, 3, and 5. Although the same videos were analysed, there are inconsistencies in reported methodology between the two papers. All dogs were trained by the owner rather than the professional trainer on the last day [usually Day 5, change in trainer not mentioned by China et al. (1)]. Dogs were on-lead for most of the training sessions and within 1 m of the trainer for 70% of the time [not mentioned by Cooper et al. (2)]. Repeated-measures statistics were used (appropriately) by Cooper et al. (2), but not by China et al. (1). Observer reliability testing for data extraction was used by Cooper et al. (2), but not by China et al. (1).

Blind data extraction occurred in both studies and is good practice, but if delivery of shock was not noticeable in the dogs' reactions (implied for the “shocked” group if the observers were truly blind), then the dogs were unlikely to have experienced “unnecessary suffering” (p. 9). *Minimum* shock levels were defined for each e-collar dog on Day 1, suggesting that shocks, if delivered at all, were mild. The training protocol was to negatively reinforce compliance on an obedience task (come or sit) through removal of electric shock or tugs on the lead followed by positive reinforcement (such as a verbal “good dog”). Such a procedure should progressively increase compliance, although there may be limits to that outcome if competing naturally reinforcing behaviours are not separately extinguished. Early research established the principle that elimination of unwanted behaviours is best achieved if the unwanted behaviour is made contingent on punishment delivered at the *maximum* acceptable level of intensity (6). Classical conditioning, involving pairing a target stimulus with an intense unpleasant experience, can eliminate approach behaviour in as little as one event (as in the kiwi example). China et al. (1) do not make use of these contingencies.

Owners reported a very high probability of the unwanted behaviour (noting that this was aggression, not obedience). Yet, in the training sessions, all dogs exhibited remarkably high compliance to obedience commands—even on the first day. The absence of baseline pre-treatment data makes it difficult to assess the effects of the training context, although aspects of it, for example, being on lead and close to a stranger in strange circumstances, were the likely cause of high overall compliance. Increasing familiarity with a strange context possibly contributed to the reduced compliance that was observed in some groups over time [Figures 4, 5 (1)]. However, the shift to a different trainer (the owner) on Day 5 also compromises both the claim that the training methodology was the same on all training days, and the interpretation of the results. Both of the key statistical results appear to rely most strongly on differences between Day 5 and the other 2 days, and those differences could be attributable to the dogs' response to the owner-as-trainer.

We have concerns about the statistical analyses. We believe that a repeated-measures analysis, as used by Cooper et al. (2), should have been used by China et al. (1) (the quoted degrees of freedom indicate that it was not), that interaction effects should have been explored (the data in Figure 4 (1) suggest a significant interaction that would have delivered the opposite result to the conclusion drawn), and that Type I errors were introduced by multiple analyses [e.g., a Bonferroni adjustment would likely have eliminated the first significant result in Table 5 (1)]. These statistical anomalies are not minor issues, as they potentially compromise or even reverse the key results in the paper.

## Conclusion

Most dogs were referred for behavioral treatment because of aggression, primarily attacks on farm animals. Most training was conducted close to sheep and chickens. Thus, the context of undesirable behaviour was mimicked, but the problem behaviors were not addressed. No background information is provided on the baseline compliance of any of the dogs with standard obedience commands, although the data for Day 1 in Figures 4, 5 (1) suggest they were high. However, attacks by dogs are unlikely to be initiated when the owner is within 1 m and the dog is on-lead. China et al.'s (1) results shed no light on the possible behavior of the dog off-lead or when the owner is absent, and therefore cannot be used as an empirical justification for removing e-collars as a technique for treating dogs with behavioural problems.

The claim about unnecessary suffering to the dog [final sentence, p. 9, (1)] was not based on any presented results and reflects a moral or welfare perspective outside the framework of the paper.

Use of intense electric shock clearly raises ethical concerns [reviewed by (7)], but when elimination of unwanted behavior is essential, e.g., because the lives of both the dog and the attacked animal are at stake, it may be morally more appropriate to use very few deliveries of a strong and effective shock, than many deliveries of weak negative reinforcement that may not eliminate the problem behavior.

As applied researchers ourselves, we applaud the researchers for their contribution to the research on e-collars. However, the research presented by China et al. (1) is, at best, a limited test of e-collars. The results are compromised by design constraints and inappropriate statistical analyses. The problem for which the dogs were referred to trainers was not directly addressed, and the real-life context in which problem behaviors occur was not replicated in the study.

## Author Contributions

RS and IM worked equally on the commentary. All authors contributed to the article and approved the submitted version.

## Conflict of Interest

The authors declare that the research was conducted in the absence of any commercial or financial relationships that could be construed as a potential conflict of interest.
